# The cost-effectiveness of a cash-based transfer, specialised nutritious food, and social and behaviour change communication intervention package to prevent undernutrition among children 6–23 months in Pakistan: A cluster randomised controlled trial

**DOI:** 10.7189/jogh.14.04186

**Published:** 2024-11-22

**Authors:** Michael N Onah, Gul Nawaz Khan, Sumra Kureishy, Jessica Bourdaire, Saskia de Pee, Cecilia Garzon, Yasir Ihtesham, Naveed Akbar, Sajid Bashir Soofi

**Affiliations:** 1 Department of Paediatrics and Child Health, Aga Khan University, Karachi, Pakistan; 2 Centre of Excellence in Women & Child Health, Aga Khan University, Karachi, Pakistan; 3 Institute of Public Policy and Administration, University of Central Asia; 4 UN World Food Programme; 5 Benazir Income Support Programme, Government of Pakistan, Pakistan

## Abstract

**Background:**

There is mixed evidence on the cost-effectiveness of cash transfers, along with food supplements and behaviour change communication interventions in improving child nutrition outcomes. To add to existing literature, we examined the cost-effectiveness of medium-quantity lipid-based nutrient supplement (LNS) and social and behaviour change communication (SBCC) messaging, separately and combined, compared to an existing unconditional cash transfers (UCT) programme in children 6–23 months of age in the district Rahim Yar Khan, Pakistan.

**Methods:**

This was a four-arm, community-based cluster randomised controlled trial. The UCT provided a quarterly sum of USD 32, the medium-quantity LNS contained a daily ration of 50 g of LNS, and the SBCC included monthly and quarterly messaging on nutrition, health, and hygiene to eligible households. Cost data were collected from a provider perspective through the review of procurement invoices and budgets, as well as interviews with stakeholders. We examined cost-effectiveness via statistically significant differences between the intervention and control arms, and estimated as cost per case of stunting, and disability-adjusted life years (DALYs) averted at six and 18 months of intervention.

**Results:**

Costs were higher for SBCC intervention combinations (UCT + SBCC and UCT + LNS + SBCC) due to high training costs for lady health workers. UCT + LNS achieved a reduction in stunting at a per-case cost of USDS 278.74 at six months and USD 897.15 at 18 months. UCT + LNS + SBCC achieved a reduction in stunting at per case cost of USD 846.48 at six months and USD 2324.58 at 18 months. The cost per DALYs averted for preventing stunting was USD 234 to USD 557.42 at six months, and USD 787.73 to USD 1537 at 18 months without discounting and age-weights.

**Conclusions:**

Although the affordability of such interventions is arguable, combining UCTs with LNS appears to be very cost-effective for reducing undernutrition and averting DALYs, while combining cash transfers with LNS and SBCC showed limited cost-effectiveness when targeting stunting.

**Registration:**

Clinicaltrials.gov: NCT03299218.

Childhood undernutrition continues to be of growing global challenge due to its immediate, mid-term, and long-term effects on human health and development, which extend from diminished cognitive development and growth faltering, to reduced educational attainment for individuals exposed to undernutrition at childhood [1–3]. In adulthood, studies have found a longer-term effect of childhood undernutrition on cardiovascular diseases, growth and education attainment, economic growth, and mental development [4–7].

Many interventions for childhood undernutrition have been developed with the aim of reducing the related incidence and burden [8] by tackling its root causes, including economic, dietary, and behavioural factors [9–12]. Some proposed and tested strategies include conditional or unconditional cash transfers, nutritional supplementation, and social and behavioural change communication [13–20]. Evidence also suggests that these interventions can be provided as packages for improved effectiveness [8,21].

In comparison to food aids, evidence suggests that cash transfers appear to be more cost-effective in improving nutritional outcomes [22–24]. However, the effectiveness and cost-effectiveness depend on conditionality (i.e. conditional or unconditional) and its specific type (education, health, etc.), as well as the mode of cash transfers (electronic or paper vouchers) [25–30]. Therefore, in contexts where cash transfer interventions exist as an integral part of government social protection initiatives, adding other interventions including nutrition supplements and social and behavioural change messaging to existing frameworks could be desirable [12,31–33]. However, evidence on the cost-effectiveness of a combination of these interventions is limited.

We designed this study to generate evidence on the cost-effectiveness of a varied package of interventions that are currently being incrementally added to an existing unconditional cash transfer programme in Pakistan. The primary objective was to compare the costs and cost-effectiveness of a four-arm intervention in which nutrition supplements such as medium-quantity lipid-based nutrient supplement (LNS) and social and behaviour change communication (SBCC) are each added to an existing unconditional cash transfer (UCT) programme or combined as an intervention package to avert cases of undernutrition. While nutrition interventions that target childhood stunting and wasting are encouraged to exceed six months due to the possibility of catch-up, estimates are presented at six and 18 months of intervention. From the cost perspective, meanwhile, the affordability of a given intervention is a significant determinant its of coverage and duration; hence for policy purposes, similar nutrition interventions that do not exceed six months should ideally conduct a follow-up after 12 months post-intervention to measure if children have relapsed to stunting status.

## METHODS

### Study setting

We conducted this study in the district of Rahim Yar Khan, located in the southern part of Punjab province, Pakistan. The district itself is predominantly agrarian; it has a population of 4.8 million people, of whom 79% live in rural areas [34]. Within the district, 13.1% of the population have access to improved sources of drinking water (piped water), 75.6% have access to improved sanitation, and 90.6% have access to electricity. Its infant mortality (56 infant deaths per 1000 live births) and under-five mortality (66 child deaths per 1000 live births) are similar to the provincial (60 infant deaths per 1000 live births, 69 child deaths per 1000 live births) and national averages (62 infant deaths per 1000 live births, 74 child deaths per 1000 live births) [35].

### Study design and participants

This was a four-arm, community-based cluster randomised controlled trial of children aged six months from the poorest households in the lowest wealth quintile, identified using Lady Health Worker (LHW) registers and Benazir Income Support Programme (BISP) beneficiary committees. We excluded children with severe acute malnutrition and/or chronic illnesses from the study and referred them to the nearest health facility for treatment. Monthly follow-up visits were conducted from the age of seven to 24 months for data collection across all study arms.

### Randomisation

**Figure 1:**
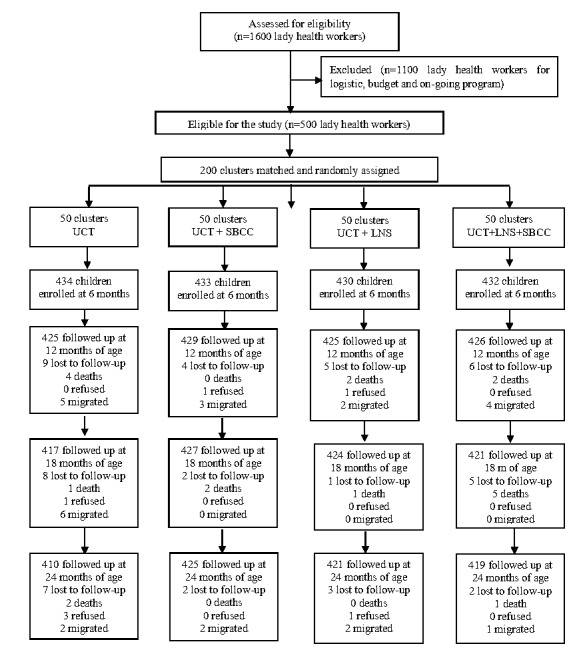
Sample size and randomisation.

The randomisation unit for delivering the intervention package was the existing LHW catchment area, each covering a population of 1000–1500 people or approximately 200 households (**Figure 1**). We arbitrarily selected three *tehsils* (Rahim Yar Khan, Sadiq Abad, and Khan Pur) in southern Punjab to facilitate safe, efficient, and effective data collection by the research team, considering distance and travel time. We employed a two-stage stratified random sampling strategy to minimise contamination risk among study arms. At the first stage, we used probability proportional to size to select union councils with higher LHW coverage, proportionate to population size. The second stage ensured equal probability of selecting LHW catchment areas and identifying an equal number of eligible children per catchment area. Out of 1600 identified LHW catchment areas, we randomly selected 200 clusters and assigned them to one of four study arms, with each arm consisting of 50 clusters. An independent statistician, not involved in the study, conducted the randomisation. Blinding of study participants and study arms was not possible for data collection teams and investigators due to their roles in supervising the provision of LNS and SBCC sessions. However, data analysts remained blinded to study participants and study arms until the final data analysis was completed.

### Sample size

The sample size was estimated to be 400 children per arm, based on a 45% baseline stunting prevalence in Rahim Yar Khan, to detect a 20% difference (with 80% power, α of 0.05, and an intra-cluster correlation of 0.0008) in stunting prevalence among children at 24 months. The assumed stunting prevalence ranged by ±5%. We calculated the coefficient of variation using this range and derived intra-cluster correlation coefficients from its formula.

Further details on study methods and statistical analysis can be found in the main study on the trial [36].

### Interventions

The intervention involved a four-arm cluster randomised controlled trial of different combinations of UCT, LNS, and SBCC (**Table 1**) delivered by our institute between May 2017 and July 2019. The trial’s aim was to assess the effectiveness of the intervention to prevent stunting in children aged 6–23 months living in households that receive the UCT. The intervention was compared with a control group of households which received routine government health services, but were ineligible to receive the UCT. The cash transfer was provided through the existing national social protection programme (the BISP) and the LNS and SBCC through LHWs and lady health supervisors (LHSs) recruited from the provincial Integrated Reproductive Maternal Newborn & Child Health and Nutrition Program.

**Table 1:** Study arm description

Control group	Lipid-based nutrient supplement (UCT + LNS)	Social and behavioural change communication (UCT + SBCC)	LNS and SBCC combinations (UCT + LNS + SBCC)
Non-BISP households who received routine government health services but were ineligible to receive the UCT.	A local product called Wawamum given to children aged 6–23 mo on a daily ration of 50 g (one sachet) to cover the recommended daily allowance of most micronutrients.	Health, nutrition and hygiene messages were provided by LHWs during routine monthly household visits.	Households received LNS and SBCC combinations.
		Community sessions were conducted with the help of a specialised picture-booklet by LHWs on a quarterly basis.	
	A total of PKR 5000 or USD 32 on quarterly basis was transferred by BISP throughout the study period.	A total of PKR 5000 or USD 32 on quarterly basis was transferred by BISP throughout the study period.	A total of PKR 5000 or USD 32 on quarterly basis was transferred by BISP throughout the study period.
434 households and children enrolled.	430 households and children enrolled.	433 households and children enrolled	432 households and children enrolled

BISP – Benazir Income Support Programme, LHW – Lady Health Worker, LNS – lipid-based nutrient supplement, SBCC – social and behaviour change communication, UCT – unconditional cash transfers, PKR – Pakistani Rupees

Children six months of age living in BISP beneficiary households (households with poverty score of less than 16.17) were eligible to participate in the intervention arms of the study, while those six months of age residing in communities served by the LHWs, but from non-BISP beneficiary households with a poverty score of 16.18–20.00 were enrolled in the control arm. Although the control group belonged to a marginally higher socioeconomic status with poverty scores, all households had similar prevalence of undernutrition at baseline.

### Cost methods

We conducted a cost-effectiveness analysis (CEA) from a provider perspective and directly related the costs collected to the procurement and distribution of the cash transfer, LNS, and SBCC given to primary caregivers during 18 months of the intervention. These costs were collected for each arm of the intervention under broad cost centres and included procurement, transport, personnel, and training costs. Specifically, we collected personnel, utilities, and administrative costs for UCT, and otherwise estimated personnel costs of LHWs and LHSs and cost of procurement and delivery of the LNS to households for LNS and personnel costs of home visits and community sessions for LHWs and LHSs, production cost of SBCC booklets, and training costs of LHWs and LHSs for SBCC.

We collected cost data by reviewing procurement invoices and budgets, and interviewing stakeholders involved in the different arms of the intervention. Costs were estimated based on the different activities involved by each arm of the intervention. We reported them as total costs per child in USD (as of October 2019, based on our own estimates) We did not include BISP transfer values (PKR 5000 or USD 32) in the cost analysis, as they are independent of operational factors. Cost data were presented as unadjusted and adjusted, where 10% of total cost was added to unadjusted costs to represent overhead costs.

### Effectiveness methods

The study outcomes were the number of cases of stunting (height-for-age z-score (HAZ)<−2) cases, and disability-adjusted life years (DALYs) averted. The CEA was carried out on stunting prevalence reduction differences between the intervention and control arm group at six and 18 months of intervention. We only focussed on statistically significant differences obtained from the impact study [36]. The trial used pairwise tests to establish significance arising from a shift in stunting status between intervention groups. The CEA utilised effectiveness estimates at six (age of 12 months) and 18 (age of 24 months) months of the interventions, while cases averted by the three interventions (i.e. UCT + LNS, UCT + SBCC, and UCT + LNS + SBCC) relative to the control group were also calculated [36].

DALYs averted were estimated for stunting only, since there were no statistically significant differences for wasting and underweight outcomes. DALYs are a way of quantifying years of healthy life lost due to illness and are made up of two components; years of life lost (YLL) due to premature mortality in the population and the years lost due to disability (YLD). Using World Health Organization (WHO) recommendations, we estimated DALYs using the formula *DALYs* = *YLL* + *YLD*. For non- discounted and non-age weighted DALYS, we estimated YLLs per the formula *YLL* = *N* × *L*, where is number of deaths and is life expectancy at the age of death. We otherwise calculated YLDs using the formula *YLD* = *P* × *DW*, where is the number of prevalent cases and is the disability weight. Afterwards, we estimated discounted and age-weighted DALYs using the Rushby and Hanson equation [37].



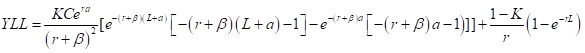



and



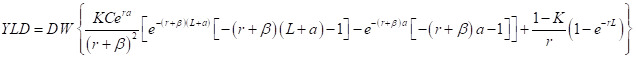



Where is the age of death in years, is the discount rate, is age-weighting constant, is the age-weighting modulation constant, and is the adjustment constant for age-weights.

We assumed the age at onset of stunting to be the average cohort age (i.e. six months) and the duration of illness to be lifelong for stunted cases reflecting previous cost-effectiveness assumptions [33]. We calculated life expectancy in Pakistan as a sex-weighted average using the local life expectancy of 69.25, calculated separately for each intervention arm [38]. For stunting, we used the disability weight of 0.002 from the Global Burden of Disease study (GBD) published in 1990 and retained in subsequent studies that have used GBD data [33,39]. The disability weight for death was 1.000. We then estimated mortality rates attributed to stunting using the 2018 under-five mortality rate; this was adjusted to exclude neonatal and infant mortality (probability of death in children aged less than one year) [40]. We converted rates to proportions of children dying over three years. We used a 3% discount rate and a 0.04 age-weight [37]. We multiplied the numbers of estimated cases by the resulting proportions to yield the expected numbers of deaths for each cause. We calculated cause-specific YLL and YLD components and summed them to estimate the number of DALYs averted for each intervention.

We conducted one-way sensitivity analyses by varying the cost and outcome variables individually over a range of probable values at 18 months of intervention. To achieve this, we utilised the 95% confidence interval (CI) of the number of non-discounted and non-age weighted DALYs averted for the low and high scenarios. When modelling costs, we assigned a plausible range based on the cost of overheads for each intervention arm specified as low (10%) or high (25%) of total costs. We did not extrapolate the estimated DALYs and costs over any number of future years or to a scaled-up population.

### Ethics statement

The ethics review committee of Aga Khan University Karachi (4572-Ped-ERC-16) and the National Bioethics Committee of Pakistan (NBC-238) gave ethical approval for our study. Informed consent was obtained from all parents or caregivers prior to recruitment, data collection, and anthropometric measurements.

## RESULTS

### Baseline characteristics

We enrolled 1745 children in the study. At baseline, all four trial arms had comparable characteristics related to mean household size, access to an improved source of drinking water, improved sanitation facility, mean age of mothers, education and occupation of mothers, body mass index (BMI) levels among mothers, mean height of mothers, education of fathers, mean height of fathers, child’s age and gender, child’s mean height and weight, prevalence of stunting and wasting, child ever breastfed and child vaccination coverage (**Table 2**) [36].

**Table 2:** Baseline characteristics of households, mothers and children by study arms*

Variables	UCT (n = 434)	UCT + SBCC (n = 433)	UCT + LNS (n = 430)	UCT + SBCC + LNS (n = 432)
Household size, x̄ (SD)	7.7 (2.5)	7.5 (2.5)	7.9 (2.5)	7.7 (2.5)
BISP poverty score, x̄ (SD)	11.32 (0.67)	12.47 (0.67)	11.00 (0.66)	11.55 (0.66)
Mother’s age in years, x̄ (SD)	28.7 (7.8)	31.0 (7.8)	28.6 (7.8)	29.6 (7.8)
Mother’s years of schooling, x̄ (SD)	1.5 (3.7)	0.9 (3.7)	1.4 (3.7)	1.0 (3.7)
Total pregnancies, x̄ (SD)	4.2 (2.6)	4.4 (2.6)	4.0 (2.6)	4.2 (2.6)
Mother’s BMI, x̄ (SD)	22.4 (3.7)	21.7 (3.8)	22.0 (3.8)	22.0 (3.8)
Mother’s height in cm, x̄ (SD)	154.2 (6.3)	155.2 (6.4)	154.1 (6.4)	153.3 (6.4)
Father’s years of schooling, x̄ (SD)	2.9 (4.3)	3.1 (4.3)	3.0 (4.3)	2.8 (4.3)
Father’s height in cm, x̄ (SD)	164.6 (6.4)	165.0 (6.6)	167.0 (6.6)	167.6 (6.5)
Child’s age in months, x̄ (SD)	6.2 (0.3)	6.3 (0.3)	6.3 (0.3)	6.2 (0.3)
Child’s length in cm, x̄ (SD)	64.4 (2.8)	64.5 (2.7)	64.1 (2.7)	63.9 (2.7)
Child’s weight in kg, x̄ (SD)	6.7 (1.3)	6.7 (1.3)	6.7 (1.3)	6.7 (1.3)
Improved water	424 (97.7)	433 (100.0)	418 (97.2)	418 (96.8)
Improved sanitation facility	313 (72.1)	254 (58.7)	323 (75.1)	175 (40.5)
Wood as fuel for cooking	355 (81.8)	270 (62.4)	278 (64.7)	360 (83.3)
Mother's occupation				
*Housewife*	392 (90.3)	429 (99.1)	381 (88.6)	421 (97.4)
*Working woman*	42 (9.7)	4 (0.9)	49 (11.4)	11 (2.5)
Mother’s BMI in kg per m^2^				
*Underweight (<18.5)*	74 (18.3)	87 (20.5)	83 (19.6)	78 (18.7)
*Normal (18.5–24.9)*	232 (57.3)	254 (59.9)	242 (57.2)	249 (59.7)
*Overweight (25–29.9)*	72 (17.8)	66 (15.6)	73 (17.3)	70 (16.8)
*Obese (≥30)*	27 (6.7)	17 (4.0)	25 (5.9)	20 (4.8)
Child’s gender				
*Male*	240 (55.3)	224 (51.7)	226 (52.6)	225 (52.1)
*Female*	194 (44.7)	209 (48.3)	204 (47.4)	207 (47.9)
Stunted	108 (24.9)	115 (26.6)	120 (27.9)	115 (26.8)
Underweight	103 (23.8)	113 (26.1)	111 (25.9)	108 (25.1)
Wasted	54 (12.5)	53 (12.2)	55 (12.8)	52 (12.1)
Ever breastfed	434 (100.0)	433 (100.0)	430 (100.0)	432 (100.0)
Breastfeeding at six months	402 (92.6)	398 (91.9)	396 (92.1)	379 (87.7)
All vaccination by six months	407 (93.8)	362 (83.6)	406 (94.4)	397 (91.9)
Illness during last two weeks				
*High grade fever*	337 (77.7)	307 (70.9)	240 (55.8)	225 (52.1)
*Diarrhoea*	248 (57.1)	169 (39.0)	167 (38.8)	90 (20.8)
*ARI*	224 (51.6)	91 (21.0)	102 (23.7)	12 (2.8)

ARI – acute respiratory infections, BMI – body mass index, BISP – Benazir Income Support Programme, LNS – lipid-based nutrient supplement, SBCC – social and behaviour change communication, UCT – unconditional cash transfers, x̄ – mean

*Proportions and means are cluster adjusted. Presented as n (%) unless specified otherwise.

### Intervention costs

The average intervention cost per child for six months of intervention was USD 22.04 for UCT + LNS, USD 47.40 for UCT + SBCC, and USD 70.54 for UCT + LNS + SBCC arm. When costs are adjusted, the cost per child increased to USD 25.87 for UCT + LNS, USD 52.44 for UCT + SBCC, and USD 77.23 for UCT + LNS + SBCC arm. The major cost driver for SBCC was training costs, which were 34% of the total cost. This was not incurred for UCT + LNS arm since the LHWs and LHSs were not required to be trained on SBCC. After 18 months of intervention or at 24 months of child age, the average intervention-unadjusted cost per child was USD 75.11 for UCT + LNS, USD 151.21 for UCT + SBCC, and USD 220.62 UCT + LNS + SBCC arm. When costs were adjusted, the cost per child increased to USD 82.62 for UCT + LNS, USD 166.33 for UCT + SBCC, and USD 210.68 for UCT + LNS + SBCC arm (**Table 3**).

**Table 3:** Average cost per child from enrollment to six and 18 mo of intervention

	Cost per child (unadjusted) in USD	Cost per child (adjusted) in USD	Cost per cohort (unadjusted) in USD	Cost per cohort (adjusted) in USD
**Cost for 6 mo of intervention (age of 12 mo)**				
UCT + LNS	22.04	25.87	9477.2	10 424.92
UCT + SBCC	47.40	52.44	20 524.2	22 576.62
UCT + LNS + SBCC	70.54	77.23	30 473.28	33 520.61
**Costs for 18 mo of intervention (age of 24 mo)**				
UCT + LNS	75.11	82.62	32 297.30	35 527.03
UCT + SBCC	151.21	166.33	65 473.93	72 021.32
UCT + LNS + SBCC	220.62	240.68	95 307.84	104 838.62

LNS – lipid-based nutrient supplement, mo – month, SBCC – social and behaviour change communication, UCT – unconditional cash transfers

### Cost-effectiveness

The cost-ffectiveness analysis showed that the intervention package resulted in a significant reduction in the prevalence of stunting (8.3%) at 12 months of age and (7.6%) at 24 months of age in the UCT+LNS arm. Similarly, 10% and 7.5% of significant reduction in the prevalence of stunting at 12 and 24 months of age was noted in the UCT + LNS + SBCC arm, respectively. No significant reduction in the prevalence of stunting was noted in UCT + SBCC arm (**Table 4**)

**Table 4:** Cost-effectiveness estimates by intervention

	UCT + LNS		UCT+SBCC		UCT+LNS+SBCC	
**Result**s	Six mo intervention (age of 12 mo)*	**18 mo intervention (age of 24 mo)**	**Six mo intervention (age of 12 mo)**	**18 mo intervention (age of 24 mo)**	**Six mo intervention (age of 12 mo)**	**18 mo intervention (age of 24 mo)**
Percentage reduction in prevalence of stunting	8.3	7.6	NS	NS	10.0	7.5
Number of cases of stunting averted	34	36			36	41
Cost per case of stunting averted (unadjusted) in USD	278.74	897.15			846.48	2324.58
Cost per case of stunting averted (adjusted) in USD	306.61	986.86			931.13	2530.27

LNS – lipid-based nutrient supplement, mo – month, NS – not significant, SBCC – social and behaviour change communication, UCT – unconditional cash transfers

*Sample size examined at six and 18 mo of intervention differed, hence prevalence reported differed as well.

After six months of intervention (at 12 months of child age), the non-discounted or non-age-weighted cost per DALY averted for preventing stunting was USD 243 for UCT + LNS, increasing to USD 557.42 when DALYs were discounted and age-weighted. For UCT + LNS + SBCC, the non-discounted and non-age-weighted cost per DALY averted for preventing stunting was USD 743.25, increasing to USD 1451.11 when DALYs were discounted and age weighted. After 18 months of intervention, the non-discounted and non-age-weighted cost per DALY averted for preventing stunting was USD 787.73 for UCT + LNS intervention arm, increasing USD 1537.97 when DALYs were discounted and age-weighted. For UCT + LNS + SBCC, the non-discounted and non-age-weighted cost per DALY averted for preventing stunting was USD 743.25, increasing to USD 1451.11 when DALYs were discounted and age weighted (**Table 5**).

**Table 5:** DALY estimates by intervention*

	Outcome	DALYs component	Six mo of intervention (age of 12 mo)	18 mo of intervention (age of 24 mo)
**Non-discounted and non-age weighted†**				
UCT + LNS	Stunting	DALYs	39 (10–44)	41 (11–54)
		Cost/DALYs averted, USD	243 (118.05–1099.10)	787.73 (509.80–2422.45)
UCT + LNS + SBCC	Stunting	DALYs	41 (8–49)	53 (17–71)
		Cost/DALYs averted, USD	743.25 (479.01–2100.10)	2888.12 (1223.10–7881.1)
**Discounted and age-weighted†**				
UCT + LNS	Stunting	DALYs	17 (15–21)	21 (15–30)
		Cost/DALYs averted, USD	557.48 (287.05–999.40)	1537.97 (669.80–2872.45)
UCT + LNS + SBCC	Stunting	DALYs	21 (12–28)	27 (16–41)
		Cost/DALYs averted, USD	1451.70 (109.21–800.90)	3529.92 (1917.40–8408.70)

DALY – disability-adjusted life years, LNS – lipid-based nutrient supplement, mo – month, SBCC – social and behaviour change communication, UCT – unconditional cash transfers

*Presented as estimate (95% CI) unless specified otherwise.

†Default values were used for the age-weighting modulating factor (1.000), constant term (0.1658), discount rate (0.03), and beta parameter for age-weighting (0.04) [39].

### Sensitivity analyses

Changes in cost per DALY averted when DALYs for each intervention arm and cost variables were varied between their maximum and minimum plausible values. Using the lower-bound and upper-bound CIs for the DALYs, there is a higher level of uncertainty compared with when overhead costs are varied. After six months of intervention, when overhead costs were varied at 10% and 25% of direct costs, the cost per DALYs averted for preventing stunting increased to USD 267.30 and USD303.76 from USD 243 base estimate for UCT + LNS. Similarly, the cost per DALYs averted for preventing stunting increased to USD 817.57 and USD 929.06 from USD 743.25 base estimate when overhead costs were varied in UCT + LNS + SBCC. After 18 months of intervention, the cost per DALYs averted for preventing stunting increased to USD 866.51 and USD 1083.14 for UCT + LNS from USD 787.73 base estimate, and to USD 3176.92 and USD 3971.16 from USD 2888.12 base estimate for UCT + LNS + SBCC arm (**Table 6**).

**Table 6:** One-way sensitivity analyses by varying overhead costs at 10% and 25% of total costs

		

	Six mo of intervention (age of 12 mo)			18 mo of intervention (age of 24 mo)		
	**Base**	**Low**	**High**	**Base**	**Low**	**High**
**UCT + LNS**						
Stunting						
*Cost in USD*	9477.2	10 424.92	11 846.50	32 297.30	35 527.03	44 408.79
*Cost/DALYs in USD*	243	267.30	303.76	787.73	866.51	1083.14
**UCT + LNS + SBCC**						
Stunting						
*Cost in USD*	30 473.28	33 520.61	38 091.60	95 307.84	104 838.62	131 048.28
*Cost/DALYs in USD*	743.25	817.57	929.06	2888.12	3176.92	3971.16

DALY – disability-adjusted life years, LNS – lipid-based nutrient supplement, mo – month, SBCC – social and behaviour change communication, UCT – unconditional cash transfers

After six months of intervention, the cost per DALYs averted for preventing stunting decreased to USD 212.39 (using upper bound CI) and increased to USD 947.72 (using lower bound CI) for UCT + LNS. Similarly, the cost per DALYs averted for preventing stunting decreased to USD 621.90 (using upper bound CI) and increased to USD 3809.16 (using lower bound CI) in UCT + LNS + SBCC. After 18 months of intervention, the cost per DALYs averted for preventing stunting decreased to USD 598.09 (using upper bound CI) and increased to USD 2936.12 (using lower bound CI) for UCT + LNS. While in UCT + LNS + SBCC arm, the cost per DALYs averted for preventing stunting decreased to USD 1342.36 (using upper bound CI) and increased to USD 5606.34 (using lower bound CI) (**Table 7**).

**Table 7:** One-way sensitivity analyses by using low-end and high-end confidence intervals of DALYs averted

	Six mo of intervention (age of 12 mo)			18 mo of intervention (age of 24 mo)		
	**Base**	**Low**	**High**	**Base**	**Low**	**High**
**UCT + LNS**						
Stunting						
*DALYs*	39	10	44	41	11	54
*Cost/DALYs in USD*	243	947.72	212.39	787.73	2936.12	598.09
**UCT + LNS + SBCC**						
Stunting						
*DALYs*	41	8	49	53	17	71
*Cost/DALYs in USD*	743.25	3809.16	621.90	1798.26	5606.34	1342.36

DALY – disability-adjusted life years, LNS – lipid-based nutrient supplement, mo – month, SBCC – social and behaviour change communication, UCT – unconditional cash transfers

## DISCUSSION

The UCT + LNS and UCT + LNS + SBCC interventions had statistically significant effects in averting cases of stunting after six and 18 months of intervention. The UCT + SBCC intervention, in turn, did not have a statistically significant effect in averting stunting at 18 months of intervention.

### Cases of stunting averted

The cost to avert stunting in UCT + LNS after six months of intervention was USD 278.74, increasing to USD 897.15 at 18 months of intervention. For UCT + LNS + SBCC, the cost to avert stunting was USD 846.49, increasing to USD 234258 at 18 months of intervention.

Cost-effectiveness studies of packages that target childhood stunting which include cash are scarce, while the amount of cash transfers, the quantity of nutrition supplements, and the content and mode of delivery of social and behavioural change communication tend to differ across existing research, further limiting the comparability of findings. A recent study in Pakistan found that the cost to avert a case of stunting to be approximately USD 1000 across three intervention arms (a double cash transfer, a standard cash transfer, and a fresh food voucher transfer) provided for six months [33]. A study from Bangladesh found that the cost of averting one infant death using an incremental nutritional supplementation intervention during pregnancy and infancy for eight months ranged between of USD 907 and USD 797 [41]. Another equity-based modelling study on data from 14 countries and one province estimated the cost per case of stunting averted from using nutrition supplementation at USD 3584 [42]. In Peru, research using a child nutrition education programme involving participative complementary feeding demonstrations, growth monitoring sessions, and an accreditation process found a cost of USD 55.16 per case of stunting averted at 18 months of intervention [43]. Modelling the implementation of a package of 10 different nutrition-specific preventive and therapeutic interventions at scale across four African countries, found the cost per case of stunting averted to range between USD 226 and USD 344 [44]. Although intervention packages, duration of intervention, and cost contexts tend to vary, these findings, alongside those of our study, suggest that nutrition-and-cash-based intervention packages could reduce the prevalence of childhood stunting.

### DALYs

At six months of intervention, the cost per DALYs averted by preventing undernutrition (stunting only) in our study ranged between USD 243 and USD 743.25 using non-discounted and non-age-weighted methods, and USD 557.43 and USD 1451.11 using age-weighted and discounted methods. Cost per DALYs averted at six months of intervention also ranged between USD 212 and USD 947.2 in the non-discounted and non-age-weighted sensitivity analyses where different parameters were varied. At 18 months of intervention, the cost per DALYs averted by preventing stunting ranged between USD 787.73 and USD 2888 using non-discounted and non-age-weighted methods, and between USD 537.94 and USD 3529 using age-weighted and discounted methods. Cost per DALYs averted at 18 months of intervention also ranged between USD 598.09 and USD 1342.36 in the non-discounted and non-age-weighted sensitivity analyses where different parameters were varied.

These variations in cost-effectiveness estimates suggest that variations in the prevalence of stunting and hence in DALYs averted would significantly affect the cost-effectiveness of these intervention packages. The intervention appears to be more cost-effective when the duration is six months. However, due to the lack of effectiveness in reducing wasting and underweight and the possibility of children developing stunting, the duration of intervention was 18 months [8].

For interpretation and use at local level decision-making, the affordability of health interventions relative to health gains is often assessed using local gross domestic product (GDP) per capita thresholds [45]. This approach suggests that interventions that are equal to or less than the prevailing GDP per capita should be considered very cost-effective, while those equal to or less than three times the prevailing GDP per capita should be considered cost-effective only. For example, when using the 2018 GDP per capita of USD 1482.4 for Pakistan, UCT + LNS was found to be very cost-effective at averting DALYs by preventing stunting at six and 18 months of intervention [38], with the UCT + LNS + SBCC being very-cost-effective at six months at 18 months in averting DALYs associated with preventing stunting. Cost-effectiveness at these thresholds is based on whether or not age discounting is applied. Our sensitivity analyses suggest that there is less uncertainty in the cost-effectiveness of the two intervention arms in averting DALYs when the cost of overheads is varied relative to when the number of DALYs averted is varied using lower and upper-bound confidence intervals.

Growing evidence suggests that these WHO thresholds do not reflect available resources for health investments and do not consider competing needs for limited resources [46,47]. Empirically derived thresholds reflecting the opportunity costs of health care spending are scarce in Pakistan [33]. A study from Malawi suggests that the threshold that reflects available resources would range between 1% and 51% of GDP per capita i.e. between USD 3 and USD 153 [48]. Therefore, many of the interventions deemed to be cost-effective will not meet the WHO CEA thresholds of three times GDP per capita. Nonetheless, there is value in using such approaches to establish cost-effectiveness of interventions due to the limited fiscal envelope available and low budgetary allocations to public health. In Pakistan, the current health expenditure stands at 2.75% of GDP (i.e. USD 39.58 per capita) [38]. In addition, public spending for social assistance (a component of social protection in Pakistan) is low overall at 0.8% of GDP in 2013, and while BISP represents the third largest expenditure in Pakistan’s public budget, spending on it is only 0.2% of the country’s GDP [49,50]. The inclusion of supplements such as LNS and/or SBCC in this context would increase the budgetary requirements, so the government would need to consider competing claims on the existing over-stretched resources due to fiscal constraints. However, the cost-effectiveness demonstrated by modelled cash-based, nutrition supplementation, and social and behavioural change messaging intervention packages is enough justification to encourage such fund allocations towards preventing undernutrition.

This study has the strength of attempting to perform a careful and complete accounting for all the costs (including personnel costs) associated with adding new products to an existing unconditional cash transfer programme (i.e. the BISP programme), regardless of which organisations or individuals bore the burden of these costs.

Cost-effectiveness studies of this type, however, have shortcomings. For example, the costing perspective limited analyses to the provider costs; a societal perspective might shed more light on cost drivers that could alter the cost-effectiveness of such interventions. There were limitations in the availability of some cost data information, and we relied on estimates from interviewing different high-level stakeholders involved in the intervention, despite some not being directly involved in routine activities of the different trial arms. As always, uncertainty exists regarding some measures of costs and effects, and this can influence cost-effectiveness estimates. For example, while attrition among children from the sample population was very low, it was not zero. Depending on the rate of loss to follow up and how it is handled in the estimates of effects, including in the percentage point reduction in prevalence, average cost-effectiveness measures might vary. Finally, CEAs rarely include consideration of social equity or other normative factors into the calculation [33,51]. Decision-makers should therefore not base decision solely on relative cost-effectiveness of intervention options.

## CONCLUSIONS

Addressing undernutrition continues to be a global concern, considering the immediate and long-term effects on economic development and health of at-risk populations. Intervention packages that combine cash transfers, nutrition supplementation such as LNS, and SBCC are potentially an important preventive strategy for reducing the prevalence of undernutrition. However, for such interventions to be scalable, government allocation is crucial in settings with high prevalence of undernutrition such as Pakistan.

This study adds to the limited literature on cost-effectiveness of interventions packages aimed at preventing stunting. Additional research on the cost and cost-effectiveness of such intervention from different perspectives including societal is needed. Furthermore, longitudinal studies are needed to provide longer term evidence of the effectiveness and perhaps the cost-effectiveness of nutrition interventions.
